# 2.A. Round table: From Ebola to COVID-19: lessons learned from health systems strengthening efforts and system shocks

**DOI:** 10.1093/eurpub/ckac129.065

**Published:** 2022-10-25

**Authors:** Organised by: Robert Koch Institute, Chair persons: Johanna Hanefeld (Germany), Hanna-Tina Fischer (Germany)

## Abstract

Five years after the devastating Ebola outbreak in West Africa, the health systems in Guinea and Sierra Leone are facing a new shock with the advent of the COVID-19 pandemic. After the Ebola outbreak, countries of the European Union including Germany played a major role in responding to Ebola in these countries and in strengthening their health systems. Guinea, Liberia and Sierra Leone received 115 million Euros combined, in terms of development assistance for health dedicated to health system strengthening and sector wide approaches. For these countries that are now facing the COVID-19 pandemic, the situation is ideal to assess how well health systems have absorbed the current pandemic, and reflect on the effectiveness of health system strengthening interventions received. The objectives of the workshop are two-fold: 1, to illustrate the extent to which health systems in Guinea and Sierra Leone were able to absorb the demands of the COVID-19 pandemic whilst continuing the ongoing delivery of essential health services; 2, to provide German and European policy makers with evidence relating to the effectiveness of investments that have been made over the last half-decade in strengthening health systems following the 2014 Ebola outbreak. To achieve this, three 10-minute presentations will be made followed by a 30-minute moderated discussion between the speakers and with the audience. In the first presentation, Dr Charbel El-Bcheraoui will provide insight into the scope and scale of health system strengthening interventions that were implemented in Guinea and Sierra Leone after the Ebola outbreak, and will describe a research project that was carried out to assess the impact of the COVID-19 pandemic on health systems in these two countries. In the second and third presentations, Prof Alexandre Delamou and Dr Abdul Mbawah evidence will be shared from Guinea and Sierra Leone respectively on the impact of COVID-19 on the health systems. Malaria will be used as a case study as is a leading cause of morbidity and mortality in both countries and it is a multifaceted disease that requires interventions through the whole spectrum of the health system, ranging from public health prevention activities like vector control, to delivery of treatment through health care services. Together, the three presentations will provide insights into the effectiveness of interventions implemented in the two West African countries to strengthen the capacity of the health systems to respond to new health emergencies. The subsequent 30-minute discussion will be chaired by Prof Johanna Hanefeld, Head of the Center for International Health Protection, Robert Koch Institute, Germany. It will focus on highlighting key issues that have been identified in the three presentations and on discussing specific recommendations that can be derived to improve health systems strengthening.

**Key messages:**

• Understanding how health systems responded to the COVID-19 pandemic illustrates what is needed now to make them more resilient to future shocks.

• Learning from what was done to strengthen health systems in past outbreaks will improve the effectiveness of future health system strengthening interventions.

**Speakers/Panellists:**

**Charbel El Bcheraoui**

Robert Koch Institute, berlin, Germany

**Alexandre Delamou**

University Gamal Abdel Nasser, Conakry, Guinea

**Abdul Mbawah**

COMAHS University of Sierra Leone, Freetown, Sierra Leone

